# Versatile Locomotion Planning and Control for Humanoid Robots

**DOI:** 10.3389/frobt.2021.712239

**Published:** 2021-08-13

**Authors:** Junhyeok Ahn, Steven Jens Jorgensen, Seung Hyeon Bang, Luis Sentis

**Affiliations:** ^1^ Human Centered Robotics Lab, Department of Mechanical Engineering, The University of Texas at Austin, Austin, TX, United States; ^2^ NASA Johnson Space Center, Houston, TX, United States; ^3^ Human Centered Robotics Lab, Department of Aerospace Engineering and Engineering Mechanics, The University of Texas at Austin, Austin, TX, United States

**Keywords:** humanoid robots, locomotion, trajectory optimization, whole body control, open source software

## Abstract

We propose a locomotion framework for bipedal robots consisting of a new motion planning method, dubbed trajectory optimization for walking robots plus (TOWR+), and a new whole-body control method, dubbed implicit hierarchical whole-body controller (IHWBC). For versatility, we consider the use of a composite rigid body (CRB) model to optimize the robot’s walking behavior. The proposed CRB model considers the floating base dynamics while accounting for the effects of the heavy distal mass of humanoids using a pre-trained centroidal inertia network. TOWR+ leverages the phase-based parameterization of its precursor, TOWR, and optimizes for base and end-effectors motions, feet contact wrenches, as well as contact timing and locations without the need to solve a complementary problem or integer program. The use of IHWBC enforces unilateral contact constraints (i.e., non-slip and non-penetration constraints) and a task hierarchy through the cost function, relaxing contact constraints and providing an implicit hierarchy between tasks. This controller provides additional flexibility and smooth task and contact transitions as applied to our 10 degree-of-freedom, line-feet biped robot DRACO. In addition, we introduce a new open-source and light-weight software architecture, dubbed planning and control (PnC), that implements and combines TOWR+ and IHWBC. PnC provides modularity, versatility, and scalability so that the provided modules can be interchanged with other motion planners and whole-body controllers and tested in an end-to-end manner. In the experimental section, we first analyze the performance of TOWR+ using various bipeds. We then demonstrate balancing behaviors on the DRACO hardware using the proposed IHWBC method. Finally, we integrate TOWR+ and IHWBC and demonstrate step-and-stop behaviors on the DRACO hardware.

## 1 Introduction

Planning dynamically feasible motions for humanoid robots is a challenging problem. One difficulty arises from the need to use contact forces to control legged locomotion while conforming to terrain elevation and friction constraints. Robustly tracking planned motions in humanoids is yet another difficulty as they need to fulfill multiple task objectives and deal with redundancy and floating base dynamics. To facilitate humanoid mobility in diverse terrains and whole-body control for trajectory tracking, this paper proposes three methods: 1) a trajectory optimization for walking robots plus (TOWR+) for versatile dynamic locomotion planning, 2) an implicit hierarchical whole-body controller (IHWBC) for effective multi-objective trajectory tracking for legged systems, and 3) a planning and control (PnC) software package to create complex mobility behaviors in diverse humanoid robots.

### 1.1 Dynamic Locomotion Planning for Legged Robots

We characterize different dynamic models used for locomotion planning purposes. Dynamic locomotion has been studied using point mass models (PM) with predefined footholds and step timings (Koolen et al., 2012; Englsberger et al., 2015; Ahn et al., 2018) and PM models’ variations to account for swing foot angular momentum (Faraji et al., 2019; Seyde et al., 2018; Ahn et al., 2020). These approaches are computationally efficient but require the contact schedule and the swing foot trajectories to be predefined by experts. This lack of automatic scheduling and foot swing trajectory optimization limits significantly their ability to realize complex behaviors.

To generate more complex and capable motions, Dai et al. (2014), Carpentier and Mansard (2018), Ponton et al. (2018) used a centroidal momentum (CM) model that projects all linkage motions onto a six-dimensional floating base, and has been used to optimize the momentum and reaction wrench trajectories with simplified feasibility assumptions about the robot kinematics and actuation. While Dai et al. (2014) incorporated linear complimentary formulations to automatically optimize contacts schedules, Kuindersma et al. (2016), Ponton et al. (2021) leveraged a separate contact planner formulated as an integer programming, to enable the optimization of contact sequences. This decoupling between the contact sequence optimization and motion optimization allows for reducing computational complexity but at a price: geometrically chosen contact sequences do not guarantee dynamical feasibility. In addition, the CM model does not include a robot’s base orientation in its state vector, which is significantly limiting for motions that involve, for instance, turning. The CM model additionally requires a full-kinematics model for further optimization of the base orientation.

Hereid et al. (2016), Mastalli et al. (2020) used a full-order (FO) model, that includes both a floating base and joint dynamics, and considered to optimize the joint state and torque trajectories of robots. Mordatch et al. (2012), Posa et al. (2013) formulated multi-contact dynamics as a contact complementary problem and automatically optimize contact interactions with the environment while simultaneously considering joint trajectories. Although these approaches demonstrate complex locomotion behaviors and discovery of novel contact sequences, they are often incapable of being used for receding horizon control because of the complexity of the FO model.

As a moderate complexity option, Winkler et al. (2018) proposed a trajectory optimization for walking robots (TOWR) algorithm using a single rigid body (SRB) model. The SRB model assumes a constant inertia embedded in a CM model and allows for further optimization of the base orientation and feet position trajectories without involving a full-kinematic model. Moreover, TOWR optimizes contact sequences and timing without solving an integer programming or complementary problems. This framework is highly versatile to discover new gait patterns and motions with minimal predefined information yet is still computationally efficient thanks to a novel phase-based parameterization. Unfortunately, the SRB model uses a constant inertia tensor for articulated robots without considering the limbs’ inertia and assumes a robot morphology based on point-feet. While these are reasonable assumptions for quadrupeds, they oversimplify the dynamics of humanoids. Table 1 summarizes some properties of various legged robots^1^, where the quadruped ANYmal, has a point-feet with a light-weighted leg, while the compared humanoids have line-feet or surface-feet with significantly heavier legs. As such, the DRACO, ATLAS, and Valkyrie humanoids have larger centroidal inertia variations across its joint ranges and require a more delicate model than the SRB model^2^.

**TABLE 1:** Summary of properties of various legged robots. We have sampled 10^5^ random configurations for all robots, computed the centroidal inertia tensor, and calculated the standard deviations for *I*_*xx*_, *I*_*yy*_, and *I*_*zz*_.

		ANYmal B	Cassie	DRACO	ATLAS	Valkyrie
Foot type		Point	Line	Line	Surface	Surface
Leg DOFs		3	5	5	6	6
Leg weight (kg)		3.40	10.76	16.27	18.11	24.76
Inertia	*I*_*xx*_	0.15	0.26	1.36	3.81	4.69
Variation	*I*_*yy*_	0.43	0.23	0.81	3.25	4.14
(kg m^2^)	*I*_*zz*_	0.44	0.22	1.37	2.86	2.41

To better handle dynamic motion planning of humanoids, this paper formulates a trajectory optimization algorithm, dubbed TOWR+, that leverages TOWR’s phase-based parameterization but is tailored to humanoid and legged robots with wrench contact constraints and non-negligible centroidal inertia variations. To be more specific, we include reaction torque trajectories on the contact surfaces and end-effector orientation trajectories in the decision variables. This extension enables to consider humanoids with line or surface contacts and allows for the use of legs or other limbs with higher degrees-of-freedom than quadrupeds. In addition, we incorporate a composite rigid body (CRB) model to take the configuration dependent centroidal inertia into account. To compute the centroidal inertia without a full-kinematic model in the optimization process, we pre-train a regressor that maps floating base and end-effector configurations to the centroidal inertia tensor. We then provide an analytic expression of the trained network and its Jacobian to the optimizer.

### 1.2 Whole-Body Control

There are various types of WBC frameworks addressing the transcription of hierarchies between tasks, and unilateral contact constraints. Task hierarchies are important in WBCs to ensure that higher priority tasks will not fail due to conflict with lower priority tasks. There have been studies that impose a *strict hierarchy* on tasks, where Sentis and Khatib (2005), Kim D. et al. (2019), Kim et al. (2020) used null space projections and Saab et al. (2013), Herzog et al. (2016) solved a hierarchical quadratic program (QP). *Strict hierarchies* are attractive because they allocate robots’ resources (i.e., actuators) to accomplish higher priority tasks first and relax the enforcement of lower priority tasks. However, these techniques require an additional mechanism to compute a smooth and continuous transition between tasks as studied in Lee et al. (2012), Kim S et al. (2019). As another line of research, Wiedebach et al. (2016), Feng et al. (2014), Kuindersma et al. (2016), Apgar et al. (2018) imposed *implicit hierarchies* by directly describing the desired task space motions in the cost function with different weighting factors. Tasks weighted with larger weights result in higher priority than tasks with smaller weights. These WBCs lose the enforcement of strict dynamically consistent guarantees on the priorities, but win on being more intuitive and providing smoother transitions than WBCs with *strict hierarchies*.

WBCs typically enforce unilateral contact constraints to prevent contact penetration and slip, which can be expressed as zero accelerations of the contact bodies. Similarly, there are approaches that model contact constraints as a *hard constraint* through a null space projection technique (Sentis and Khatib, 2005; Saab et al., 2013; Kim et al., 2018) or equality constraint (Kuindersma et al., 2016; Koolen et al., 2016; Herzog et al., 2016). Although such approaches model exact unilateral constraints, they generate discontinuous torques during contact switches. As an alternative, Feng et al. (2014), Kim D. et al. (2019), Kim et al. (2020), Wiedebach et al. (2016), Apgar et al. (2018) modeled contact constraint as *soft constraint* by minimizing contact accelerations using weighting factors in the cost function. This allows for smooth contact switching through continuous interpolation of the weighting factors.

WBCs provide multi degrees-of-freedom control and are often integrated with a joint-level controller. For examples, Herzog et al. (2016), Kim et al. (2018) integrated their WBCs with a joint-level torque controller, while Feng et al. (2014), Kim et al. (2020) incorporated a joint-level position controller to handle model mismatch and provide stiff position behaviors. However, Feng et al. (2014), Kim et al. (2020) solved an inverse kinematics problem and compute joint kinematic targets, which can result in inconsistencies between the desired joint position commands and the dynamically consistent joint movements. To resolve this issue, Kuindersma et al. (2016), Koolen et al. (2016) instead integrated joint accelerations to obtain joint position commands.

In this paper we devise a new WBC framework, dubbed IHWBC. It employs an *implicit hierarchy* between multiple tasks and formulates unilateral contacts using *soft constraints* to provide continuous task and contact transitions, as well as more versatile task allocation. In addition to tracking the desired motion tasks, IHWBC tracks desired reaction wrench commands provided by motion planners and computes joint torque commands. It also computes joint position and velocity commands by integrating joint acceleration, which can then be fed to low-level joint position controllers. Although each individual feature embodied in IHWBC has been previously proposed in other WBC frameworks, this is the first time that we combine the best of all into a single IHWBC framework with an emphasis on versatility and ease of use for biped robots and humanoids.

**FIGURE 1:**
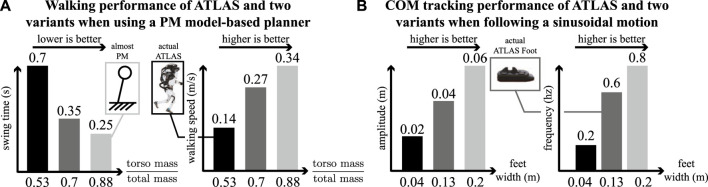
Sensitivity analysis on walking and COM tracking performance based on the robot’s mass distribution and feet size. **(A)** We created two variations of ATLAS by reducing its extremities masses and evaluated the resulting walking performance using a PM model-based planner (Englsberger et al. (2015)). As the robot’s mass distribution resembles a PM model (i.e., its distal mass becomes negligible), the robot is able to achieve faster walking speeds. **(B)** Similarly, we created two variations of ATLAS by changing its feet width and evaluated its COM tracking performance during movements in the double support stance. As the robot’s feet get wider, it’s able to track more aggressive sinusoidal trajectories.

In our previous work (Ahn et al., 2019; Kim et al., 2020), we successfully achieved unsupported dynamic stepping with DRACO using a PM model-based reactive footstep planner and a projection-based WBC. However, the dynamic stability required continuous stepping without the ability to halt, which is not suitable for loco-manipulation tasks that require the robot to have zero velocity at the end of each movement (Jorgensen et al., 2020). Unlike other bipeds, DRACO has line-feet provided by five degrees-of-freedom per leg, and non-negligible mass distribution of its limbs. While similar to Cassie, DRACO has heavier legs and has up to 5x inertial variations as summarized in Table 1. For precise walking control of DRACO, we need to consider the leg’s inertia and its angular momentum during fast swinging motions as reported by Kim et al. (2020), Wiedebach et al. (2016). In other words, we need to use an accurate model that can captures DRACO’s heavy distal mass in motion planning as Faraji et al. (2019), Seyde et al. (2018) used a three-linear-pendulum and a lumped mass model, respectively. To further support our claims, Figure 1 shows how a robot’s mass distribution and foot size affect the performance of its walking and control. Taking into consideration all of this, we integrate the proposed TOWR+ planner and IHWBC controller and demonstrate it in the DRACO biped robot to balance and stop at every step.

### 1.3 Open-Source Software Framework

Regarding locomotion planning, Crocoddyl (Mastalli et al., 2020), the multi-contact locomotion planning framework described in (Carpentier and Mansard, 2018), TOWR (Winkler et al., 2018), the kino-dynamic optimization framework described in (Ponton et al., 2021), and the inverted pendulum-based walking patter generator described in (Caron et al., 2020) have been released open-source and maintained. Regarding WBC, the task space inverse dynamics framework described in (Del Prete et al., 2016), OpenSoT (Rocchi et al., 2015), mc_rtc (Bolotnikova et al., 2020), and mim_control (Grimminger et al., 2020) have been published open-source. The packages above focus either on locomotion planning or WBC but not on both. Therefore, these packages are not self-contained in that they require external libraries to fully plan and control humanoid robots. On the other hand, IHMC’s open-robotics-software (IHMCRobotics, 2018) and MIT’s Cheetah-software (Kim S et al., 2019) included both planning and WBC modules together and provide an end-to-end testing environment.

In this paper, we provide a software architecture, dubbed PnC, that implements our proposed TOWR+ motion planner and the IHWBC feedback controller. PnC implements the building blocks with sufficient abstraction of the tasks and constraints, such that it can be used with IHWBC but also with other WBC controllers or motion planners from third parties. Our software package contains various important modules such as physics simulators, a rigid body dynamics library, motion planners, and whole-body controllers so that users can connect their own motion planner or controller and evaluate it in an end-to-end manner with minimal external dependencies. Another unique feature of PnC is that we provide it in two different languages: a C++ version^3^ for those who are interested in the real-time performance, and a Python version^4^ for those who want to simply prototype and test their ideas.

### 1.4 Contribution

We highlight the key contributions of the paper:

• We propose a motion planning framework, dubbed TOWR+, that employs a CRB model and efficiently solves for robot body motions, end-effector motions and contact wrenches, and contact sequences, timings, positions and orientations. The CRB model is a simplified floating base model but is informative enough to consider the inertia effects of heavy robots limbs for planning thanks to the use of centroidal inertia network. We demonstrate various locomotion behaviors including a multi-contact case on multiple humanoid robots using TOWR+ with thorough analysis. We demonstrate that TOWR+ is able to discover arm motions to shape the robot’s composite inertia while walking, which is a unique capability that other simplified model-based planners haven’t achieved.
• We propose a whole-body controller, dubbed IHWBC, that considers an *implicit hierarchy* between tasks and *soft constraints* to account for unilateral contacts. IHWBC tracks the desired reaction wrench commands from our planner and computes joint torque commands. It also computes joint kinematic commands by integrating joint accelerations, which can be forwarded to low-level joint controllers. IHWBC allows smooth task and contact transitions, as well as a flexible task management, which is an important feature to control humanoid robots. We demonstrate experimental results on DRACO performing lateral swinging motion of its center of mass (COM) in double support phase by following a sinusoidal trajectory using IHWBC.
• We provide a lightweight and open-source software package, dubbed PnC, that integrates planning and control modules altogether, enabling an end-to-end evaluation environment with a focus on modularity, flexibility, and generality. The software package includes TOWR+ and IHWBC, but other algorithms can also be seamlessly integrated in the framework.
• Finally, we pair our TOWR+ and IHWBC components and evaluate locomotion behaviors under unknown disturbances in a physics simulator. We also experimentally demonstrate that DRACO can take a step and stop with zero velocity by capturing the full floating base state using our TOWR+ and IHWBC. To the best of our knowledge, this zero-velocity capture behavior on a line-foot biped with significant leg mass and centroidal inertia variations has not been achieved with simplified model-based planners and WBCs.

## 2 TOWR+

**FIGURE 2:**
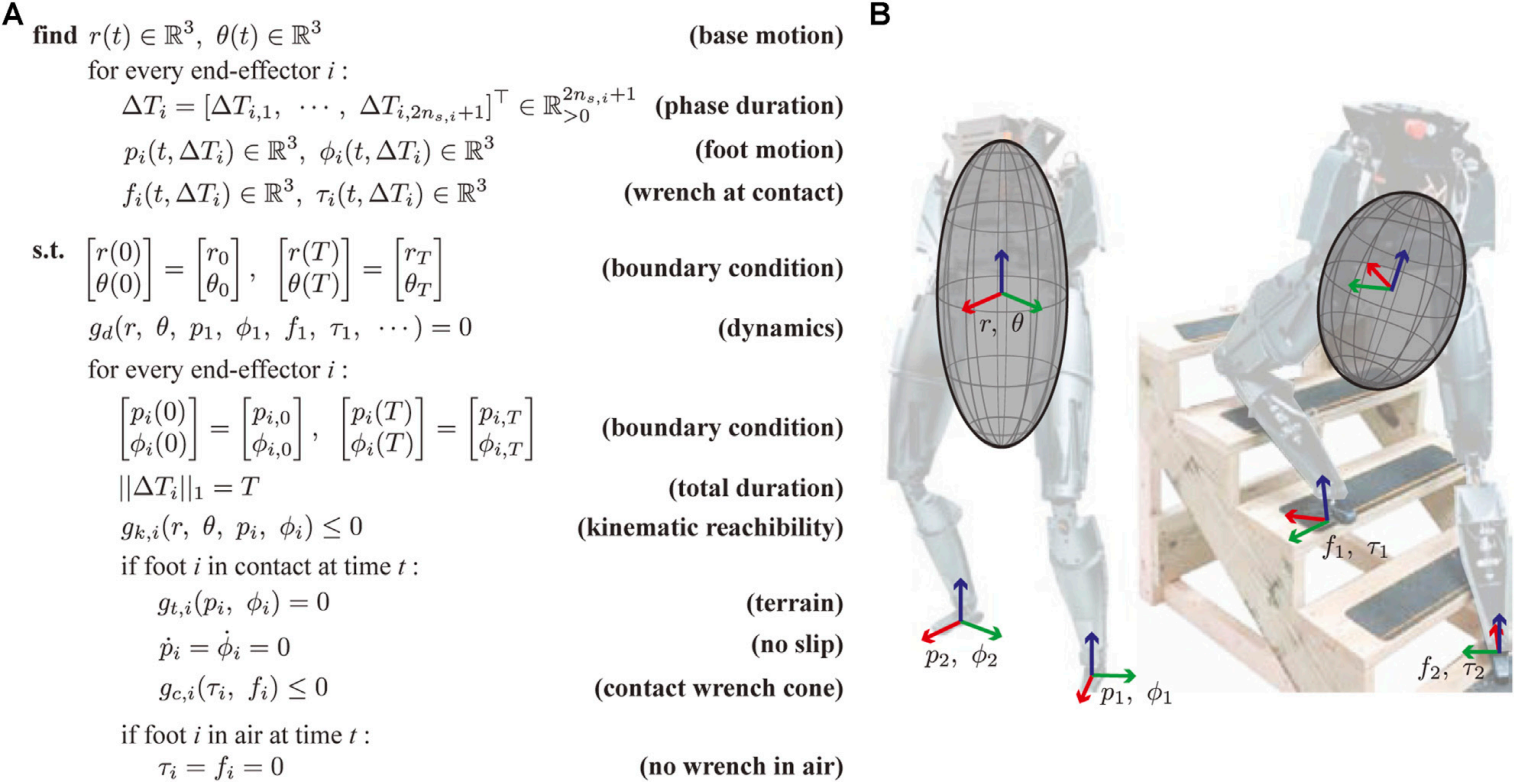
**(A)** The TOWR+ locomotion planning algorithm is presented. **(B)** Illustrative examples of the CRB model are shown for two different biped robot configurations. The configuration dependent centroidal inertias of the robot are depicted as ellipsoids.

We propose a new motion planning framework dubbed TOWR+ for humanoids by leveraging the previous study, called TOWR, by Winkler et al. (2018). Our TOWR+ motion planning framework is shown in Figure 2. Similarly to TOWR, TOWR+ takes initial and final robot body states, the desired duration of the locomotion trajectory $T\in R_{>0}$, the number of steps for each foot to reach the final robot state $n_{s,i}\in N$, and the terrain elevation as an input. TOWR+ uses this information to find the COM trajectory $r\left(t\right)\in R^{3}$, base orientation $\theta \left(t\right)\in R^{3}$, and feet positions $p_{i}\left(t\right)\in R^{3}$, orientations $\phi_{i}\left(t\right)\in R^{3}$, reaction forces $f_{i}\left(t\right)\in R^{3}$, and torques $\tau_{i}\left(t\right)\in R^{3}$. Our algorithm automatically discovers appropriate gait schedules as defined by a phase duration vector $\Delta T_{i}\in R_{>0}^{2n_{s,i}+1}$. Since each foot performs *n*_*s*,*i*_ steps which includes a swing and a contact phase, the total number of contact phases for the foot is 2*n*_*s*,*i*_ + 1. To ensure feasible motions, we employ a CRB model and simplified kinematic bounds for the feet positions. In addition, we impose additional constraints on the feet motion and contact wrenches to ensure that there is no slip on the feet and that wrenches are produced only while in contact. The decision variables and the constraints described in Figure 2 are expressed in Cartesian space for ease of use. By extending TOWR we benefit by its computational advantages compared to other hybrid control methods that rely on complex integer programming or complementarity constraint specifications.

### 2.1 Phase-Based Parameterization

**FIGURE 3:**
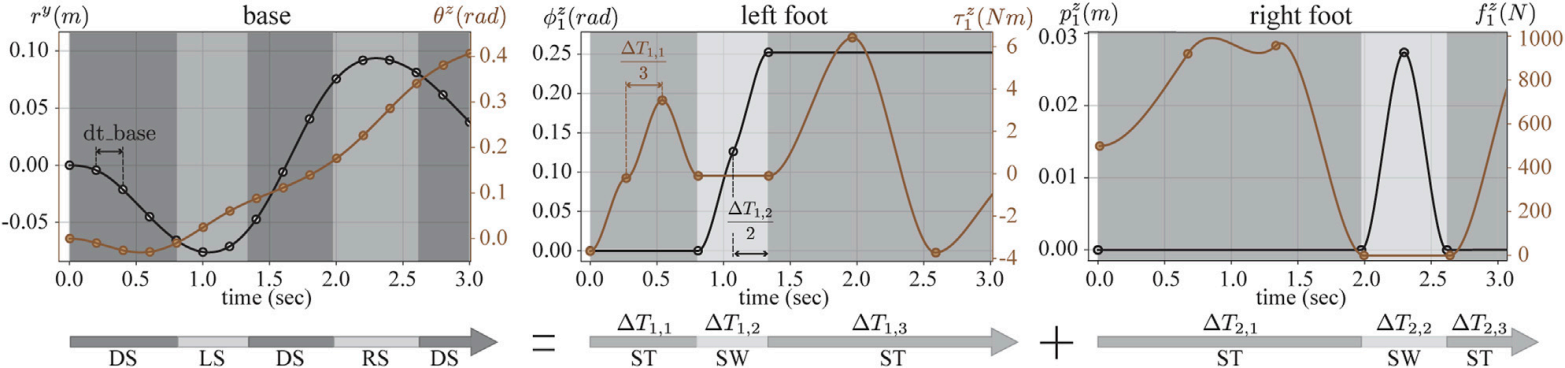
Spline trajectories for various processes are illustrated. The optimization nodes are represented by dots and the associated slopes at these positions. Cubic Hermite polynomials connect two consecutive nodes and define spline trajectories. The process trajectories are parameterized by their boundary values at the collocation nodes along their interval durations. The base motion trajectories are discretized with a fixed timestep dt_base, whereas the feet trajectories and contact wrenches are discretized with a fixed number of polynomials per phase (two for the swing trajectory, and three for the stance wrenches), whose duration is also an optimization variable. Below, three timing variables per foot are shown, where ST and SW stand for the stance and swing phase. The combination of feet phases determine the gait sequence and schedules, where DS, LS, and RS stand for double support phase, left foot swing phase, and right foot swing phase, respectively. Note that TOWR+ optimizes 6 dimensional motions (*p*_*i*_, *ϕ*_*i*_) and wrenches (*f*_*t*_, *τ*_*i*_) for each end-effector, but only few variables are illustrated here.

Similar to TOWR, we discretize the problem for numerical efficiency and we employ the above collocation method. We compose feet motions and wrench trajectories using multiple cubic Hermite polynomials, where each polynomial is defined by its duration and the positions and derivatives at the start and end of each node^5^. For instance, we discretize the base motion trajectories with a fixed timestep and parameterize their values and derivatives to solve the collocation problem. Along the base trajectories, we enforce continuous accelerations at the junction between polynomials, thus preventing sudden jumps on the contact points. As for foot motion trajectories, we use a predefined fixed number of polynomials during the swing phase and a constant position during the stance phase (since the feet are not moving). We also use a predefined fixed number of polynomials to compose contact wrench trajectories during the stance phase and use a zero wrench value during the swing phase. These parameterizations ensure that the feet only move during the swing phase and only generate reaction wrenches during the stance phase. We further constrain this processes to make sure that the feet motions and their wrench profiles are smooth and continuously differentiable during junctions. The duration of each phase is changed based on the optimized phase duration Δ*T*_*i*_. Therefore, the duration of each polynomial during feet motions and contacts is automatically determined through the optimization process, whereas the polynomial duration for base motions is fixed in advance. Figure 3 shows an example of spline trajectories for some of the decision processes with their phase durations.

### 2.2 Robot Model

Here, we discuss a kinematic model for reachability and a new CRB model based on the use of a centroidal inertia network.

Kinematic model: We consider the relative distance between the robot’s base and its feet to approximate the kinematic reachability constraint. Similarly to TOWR, we define the limits of the feet workspaces using cubes centered at nominal positions on each foot. As an extension in TOWR+, we further project each foot’s heading (i.e., the foot frame’s *x*-axis shown in Figure 2) onto the transverse plane (i.e., the base frame’s *x*-*y* plane) and keep the angle between the projected foot heading and the base heading (i.e., base frame’s *x*-axis) smaller than a threshold. Thus, we express the kinematic reachability constraint for the foot *i*, *g*_*k*,*i*_ (*r*, *θ*, *p*_*i*_, *ϕ*_*i*_) ≤ 0 as:

$$\begin{array}{c} |\underset{†}{\underset{︸}{R_{b}^{w}\left(\theta \left(t\right)\right)\left[p_{i}\left(t\right)-r\left(t\right)\right]}}-{\bar{p}}_{i}|\leq l_{max},\\ |∠\left(\left[\begin{array}{ccc} 1 & 0 & 0\\ 0 & 1 & 0 \end{array}\right]\underset{‡}{\underset{︸}{R_{ee_{i}}^{b}\left(\theta \left(t\right),\phi_{i}\left(t\right)\right)\left[\begin{array}{c} 1\\ 0\\ 0 \end{array}\right]}},\;\left[\begin{array}{c} 1\\ 0 \end{array}\right]\right)|\leq \theta_{max}, \end{array}$$ (1)

where $R_{b}^{w}$ is the rotation matrix of the base frame from the world frame, $R_{ee_{i}}^{b}$ is the rotation matrix of the *i*th foot frame from the base frame, and ${\bar{p}}_{i}$ represents the nominal position of the *i*th foot from the base frame. ∠(⋅, ⋅) measures the angle between two vectors. $l_{max}\in R^{3}$ is the maximum deviation length vector from the nominal foot position, and $\theta_{max}\in R$ is the maximum deviation angle of the projected foot heading from the base heading. Note that the expression, †, represents the foot position relative to the base frame with respect to the base frame. The quantity, ‡, denotes the foot heading (i.e., *x*-axis) with respect to the base frame, which will then be projected onto the transverse plane. These kinematic constraints are enforced at sampled states while performing the trajectory optimization process.

Centroidal inertia network: The main motivation for incorporating a centroidal inertia along with the floating base model is to properly account for the heavy distal mass of most humanoid robots. However, considering a centroidal inertia with a floating base model is non-trivial because the centroidal inertia is a function of the robot’s configuration (i.e., joint positions). To avoid solving an expensive inverse kinematic problem using the full kinematic model of a robot during the optimization process, we pre-train a centroidal inertia network that approximates the centroidal inertia tensor with respect to the base frame when the base and end-effectors configurations are given as an input with the expressions:

$${\left[\begin{array}{cccccc} I_{xx} & I_{xy} & I_{xz} & I_{yy} & I_{yz} & I_{zz} \end{array}\right]}^{⊤}=I_{b}\left(r,\;\theta ,\;p_{1},\;\phi_{1},\;\cdots \;,\;p_{n_{ee}},\phi_{n_{ee}}\right),$$ (2)

where I_*_’s are the inertia quantities at the base frame and $I_{b}:R^{6}\times R^{6\times n_{ee}}\mapsto R^{6}$ is the regressor function. The quantities *r* and *θ* represent the robot’s base configuration and *p*_*i*_ and *ϕ*_*i*_ describe the *i*th end-effector configuration. One of the benefits of our composite centroidal inertia network is that it is possible to provide an analytic expression of the regressor *I*_*b*_ and its jacobian to the optimization solver, whereas inverse kinematic approaches do not provide analytic mappings.

**FIGURE 4:**
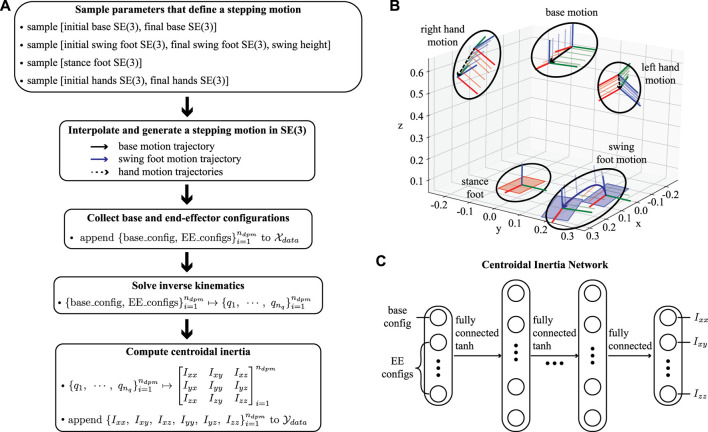
**(A)** This flowchart summarizes the data generation pipeline. **(B)** An example of a stepping motion in Cartesian space is depicted with randomly sampled initial and final configurations of the base and end-effectors. The sequence of frames for each Cartesian frame depicts our interpolation process, for instance using six samples per trajectory. **(C)** The centroidal inertia network is composed of a multi-layer perceptron that takes the base and the end-effector configuration as inputs and approximates the inertia quantities as an output.

For efficient training, we collect data from random sequences of stepping motions created using a few variables. For instance, we randomly sample initial and final configurations of the base, hands, and swinging foot, as well as stance foot configurations and swing height positions. Then, we interpolate these initial and final configurations to generate a one-step motion using Cartesian frame samples. For all these interpolated trajectories, we collect *n*_*dpm*_ samples of the base and end-effectors configurations, solve full-body inverse kinematics to compute the robot’s joint configurations along the trajectories, and compute centroidal inertia tensors. Repeating this procedure, we construct an input dataset using the base and end-effector configurations and label it using the exact centroidal inertia quantities. We then use this dataset to train the centroidal inertia network. The overall data generation pipeline and network structure are illustrated in Figure 4.

Composite rigid body model: Finally, we use the pre-trained centroidal inertia network above during the optimization process to enforce dynamic constraints. The base frame acceleration is defined by:

$$\begin{array}{cc} m\ddot{r}\left(t\right)=\overset{n_{ee}}{\underset{i=1}{\sum }}f_{i}\left(t\right)-mg,\\ I_{w}\dot{w}\left(t\right)+w\left(t\right)\times I_{w}w\left(t\right) & =\overset{n_{ee}}{\underset{i=1}{\sum }}f_{i}\left(t\right)\times \left(r\left(t\right)-p_{i}\left(t\right)\right)+\tau_{i}, \end{array}$$ (3)

where *m* is the mass of the robot, *n*_*ee*_ is the number of end-effectors, and *g* is the gravity vector. *I*_*w*_ is the centroidal inertia tensor expressed in world frame, which is efficiently predicted by the centroidal inertia network. Note that the centroidal inertia network, *I*_*b*_ predicts the output with respect to the base frame, and we apply a transformation to express it in the world frame. *w*(*t*) represents the angular velocity and can be calculated using the Euler angle rates $\dot{\theta }\left(t\right)$. We enforce the dynamic constraint at regular time intervals along the search.

### 2.3 Contact Model

As stated earlier, we use a phase-based parameterization to enforce no slip conditions and ensure zero contact wrenches while the foot is in the air. We optimize the duration of the polynomials, which determine the contact sequence and timings. For each foot’s contact phase, we additionally consider the contact wrench cone constraint and terrain elevation constraint. The contact wrench cone constraint, $g_{c,i}\left(\tau_{i},f_{i}\right)=U_{i}{\left[\tau_{i}\left(t\right),f_{i}\left(t\right)\right]}^{⊤}\leq 0$, where $U_{i}\in R^{16\times 6}$, ensures Coulomb friction fulfillment on the resultant force, the center of pressure of the foot being inside the support area, and the bounds on the yaw torque being fulfilled (Caron et al., 2015). The terrain elevation constraint enforces that the entire foot is in contact with the ground using the expressions:

$$\begin{array}{c} \left[\begin{array}{ccc} 0 & 0 & 1 \end{array}\right]p_{i}\left(t\right)=h_{\text{terrain}}\left(\left[\begin{array}{ccc} 1 & 0 & 0\\ 0 & 1 & 0 \end{array}\right]p_{i}\left(t\right)\right),\\ R_{ee_{i}}^{w}\left(\phi_{i}\left(t\right)\right)\left[\begin{array}{c} 0\\ 0\\ 1 \end{array}\right]=n_{\text{terrain}}\left(\left[\begin{array}{ccc} 1 & 0 & 0\\ 0 & 1 & 0 \end{array}\right]p_{i}\left(t\right)\right), \end{array}$$ (4)

where $R_{ee_{i}}^{w}$ represents the rotation matrix of the *i*th foot from the world frame. $h_{\text{terrain}}:R^{2}\mapsto R$ is a function that receives a two dimension location of the terrain map as an input and returns its height (elevation), and $n_{\text{terrain}}:R^{2}\mapsto R^{3}$ takes the same input and returns the normal vector of the terrain. Physically, these constraints ensure that the height of the foot matches the ground height and the contact surface is perpendicular to the terrain normal vector. Note that the contact wrench cone constraints and terrain elevation constraints are only enforced when the foot is in the contact phase and not enforced during the swing phase.

## 3 Implicit Hierarchical Whole-Body Control

We propose a new whole-body control framework, IHWBC, that employs an *implicit hierarchy* of tasks and *soft constraints* to handle unilateral contacts. This formulation enables smooth task and contact transitions, as well as flexible task management to be used in diverse legged and humanoid robots. Given desired task space objectives ${\ddot{x}}_{i}^{d}$, and a stack of desired reaction forces $f_{r}^{d}$, the goal is to find instantaneous joint accelerations $\ddot{q}$ and dynamically consistent reaction wrenches *f*_*r*_, that satisfy the robot’s kinematics and dynamics constraints. The optimal solutions $\ddot{q}*$ and $f_{r}^{*}$ are then used to compute desired joint torques *τ* using forward dynamics. The joint accelerations $\ddot{q}*$ are subsequently integrated to obtain desired joint velocities and positions that are then sent to a low-level joint controller in the robot. As noted by Feng et al. (2014), care must be taken when integrating joint accelerations to prevent instability problems, such as wind-up. We follow the integration approaches described in IHMCRobotics (2018), Jorgensen (2020). Our IHWBC is formulated as a linear constrained QP:

$$\begin{array}{ll} \underset{q¨,f_{r}}{\text{min}} & \overset{n}{\underset{i=1}{\sum }}w_{i}\|J_{i}\ddot{q}+{\dot{J}}_{i}{\dot{q}}_{m}-{\ddot{x}}_{i}^{d}{\|}^{2}+w_{f_{r}}\|f_{r}^{d}-f_{r}{\|}^{2}+\lambda_{q}\|\ddot{q}{\|}^{2}+\lambda_{f_{r}}\|f_{r}{\|}^{2} \end{array}$$ (5A)

$$\begin{array}{ll} \text{s.t.} & \;S_{f}\left(A\ddot{q}+b+g-J_{c}^{⊤}f_{r}\right)=0, \end{array}$$ (5B)

$$\begin{array}{ll} & \;Uf_{r}\geq 0, \end{array}$$ (5C)

$$\begin{array}{ll} & \;S_{r}f_{r}\leq f_{r}^{\text{max}}, \end{array}$$ (5D)

$$\begin{array}{ll} & \;{\ddot{q}}_{\text{min}}\leq \ddot{q}\leq {\ddot{q}}_{\text{max}}, \end{array}$$ (5E)

$$\begin{array}{ll} & \;\tau_{\text{min}}\leq S_{a}\left(A\ddot{q}+b+g-J_{c}^{⊤}f_{r}\right)\leq \tau_{\text{max}}. \end{array}$$ (5F)

We now discuss the individual terms of the above optimization.

Task space acceleration objectives: The first term in Eq. 5A describes task space acceleration objectives. The *i*th task space acceleration, ${\ddot{x}}_{i}^{d}$, can be computed using simple task space PD feedback, i.e., ${\ddot{x}}_{i}^{d}=k_{p,i}\left(x_{i}^{d}-x_{i}\right)+k_{d,i}\left({\dot{x}}_{i}^{d}-{\dot{x}}_{i}\right)$, where, *k*_*p*,*i*_ and *k*_*d*,*i*_ are PD gains. The desired task space position and velocity, $x_{i}^{d}$ and ${\dot{x}}_{i}^{d}$, are provided by TOWR+ or other motion planners, and the measured position and velocity, i.e., *x*_*i*_ and ${\dot{x}}_{i}$, is obtained *via* forward kinematics from joint and IMU sensor data. ${\dot{q}}_{m}$ corresponds to measured joint velocities. Each task has a priority weight, *w*_*i*_, and uses a task Jacobian *J*_*i*_ to establish a correspondence with generalize displacements. Unilateral contact constraints are accounted for by penalizing contact accelerations through assigning high values to their weights. Our strategy to incorporate non-slip constraints is to set the desired positions and velocities as the current ones (i.e., $x_{i}^{d}=x_{i}$ and ${\dot{x}}_{i}^{d}={\dot{x}}_{i}$). Placing a high weight value on contact tasks is akin to projecting lower priority tasks in the contact null space. In summary, our new IHWBC imposes an *implicit hierarchy* between tasks through the use of task weights and provides *soft constraints* to handle contacts.

Smooth task and contact transitions: Using IHWBC, we guarantee smooth task transitions by changing the relative weights *w*_*i*_ for all tasks *i*. To transition from an old task weight, $w_{i}^{\text{old}}$, to a target task weight, $w_{i}^{\text{target}}$, we perform the following linear interpolation:

$$w_{i}^{\text{update}}=\left(1-s\left(t\right)\right)w_{i}^{\text{old}}+s\left(t\right)w_{i}^{\text{target}}$$ (6)

where s:R↦[0,1] is a monotonic scalar function of time, *t*, whose output goes from 0 to 1. For instance, given a task transition time, *T*_transition_, *s*(*t*) is defined as:

$$s\left(t\right)=\frac{t}{T_{\text{transition}}}.$$ (7)

Desired reaction wrenches: The next term in the cost function, Eq. 5A, tracks desired reaction forces, $f_{r}^{d}$. A scalar weight $w_{f_{r}}$ is provided to indicate the relative priority of the force tracking task compared to tracking task space acceleration tasks. This reaction force tracking capability is used to track forces provided by motion planners, such as feet reaction forces or desired manipulation forces.

Regularization and smoothing terms: Regularization costs *λ*_*q*_ and *λ*_*f*_ in Eq. 5A are incorporated to the decision variables, $\ddot{q}$ and *f*_*r*_, respectively, to ensure that the QP cost terms are positive definite as certain configurations, *q*, can cause task Jacobians, *J*_*i*_, to be near singularities. Without these regularization terms, the QP can encounter numerical issues as some QP solvers require the quadratic cost matrix to be strictly convex (Goldfarb and Idnani, 1983; Turlach and Weingessel, 2011).

Dynamics constraint on floating base: The equality constraint Eq. 5B enforces floating base dynamics, where *S*_*f*_ is a selection matrix that extracts the first six rows of the generalized dynamics equation. *A*, *b*, *g*, and *J*_*c*_ are the mass matrix, centrifugal and coriolis force, gravity vector, and the concatenation of contact Jacobians, respectively.

Contact wrench cone and maximum force constraints: The constraints defined by Eqs 5C,D are the contact wrench cone and maximum force constraints, respectively. The matrix *U* changes depending on the contact type. Typically, the types of contacts include point, line, or surface contacts (Park and Khatib, 2008). The matrix *S*_*r*_ is a selection matrix used to bound a specific reaction force to a maximum value described by the maximum force vector $f_{r}^{\text{max}}$. In practice, we select the normal direction of the reaction forces and bound them from above while bounding the tangential force directions using the cone constraints. When a robot makes or breaks contact, we smoothly increase or decrease the upper bound so that the robot has smooth transitions. Note that for each non-slip contact constraint that is defined, we need to incorporate a position task defined at the contact frame to ensure near zero accelerations.

Kinematic constraints: We also specify minimum and maximum joint acceleration limits as shown in Eq. 5E. Alternatively, joint acceleration bounds can be enforced using position and velocity limits. For instance, assuming an integration time of Δ*t* and using Euler integration, the acceleration limits are bounded by the joint position and velocity limits as:

$$\begin{array}{c} {\dot{q}}_{\text{min}}\leq \ddot{q}\Delta t+{\dot{q}}_{m}\leq {\dot{q}}_{\text{max}},\\ q_{\text{min}}\leq \frac{1}{2}\ddot{q}\Delta t^{2}+{\dot{q}}_{m}\Delta t+q_{m}\leq q_{\text{max}}, \end{array}$$ (8)

where ${\dot{q}}_{m}$ and *q*_*m*_ are the measured joint velocity and position, respectively. A conservative limit can also be enforced by over-estimating the integration time Δ*t*. To account for all possible joint and velocity limits expressed as joint acceleration constraints, the reader can refer to Del Prete (2018).

Torque limits: Actuator saturation constraints are defined in Eq. 5F, where *S*_*a*_ is a selection matrix that extracts the bottom rows of the generalized dynamics equation corresponding to actuated joints. Once the QP solution ${\ddot{q}}^{*}$ and $f_{r}^{*}$ are obtained, the optimal joint torque command can be obtained using:

$$\tau^{\text{cmd}}=S_{a}\left(A{\ddot{q}}^{*}+b+g-J_{c}^{⊤}f_{r}^{*}\right).$$ (9)

Using the integration schemes described in IHMCRobotics (2018), Jorgensen (2020) we then compute *q* ^cmd^ and ${\dot{q}}^{\text{cmd}}$, which will then be executed by a low-level joint position controller. The torque input for each joint can be computed as:

$$\tau^{\text{joint}}=\tau^{\text{cmd}}+k_{p}\left(q^{\text{cmd}}-q_{m}\right)+k_{d}\left({\dot{q}}^{\text{cmd}}-{\dot{q}}_{m}\right),$$ (10)

where *k*_*p*_ and *k*_*d*_ are the PD gains of the low-level controller.

## 4 PnC Software Architecture

**FIGURE 5:**
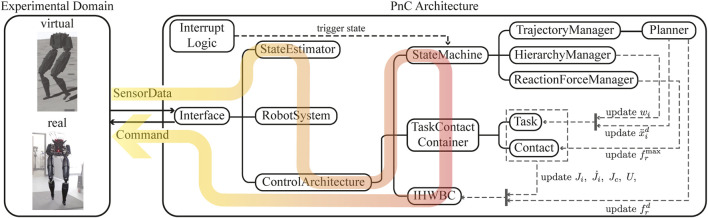
The PnC software architecture is presented with the computation flow and the individual class objects. PnC communicates with a physics simulator or the real robot hardware *via* the Interface class by receiving the robot’s information and returning a command. Internally, PnC estimates the robot’s states, and solves forward kinematics and dynamics using a rigid body dynamics library. Manager blocks update the task hierarchy weights, desired task accelerations, and the reaction force upper bounds based on finite state machines, which will then be used in the IHWBC module to compute the target joint positions, velocities, and torque commands.

We introduce a new open-source and light-weight software architecture, dubbed PnC, that implements and combines TOWR+ and IHWBC with control tools to design new behaviors. The main goal of PnC is to provide various modules for planning, whole-body control, simulation, rigid body dynamics, and utilities, such that they can be executed with minimal external dependencies. Figure 5 shows the overall PnC architecture described in more detail below.

Interface: PnC can be seamlessly employed for real-time experiments or virtual simulations. The communication is handled by the Interface class which takes SensorData inputs and returns Command as computed by PnC. Here, SensorData contains joint encoder measurements, force and torque data, IMU data, and camera data, and Command consists of desired joint positions, velocities, and torques to be applied to the robot. This computational cycle takes place at every servo loop for simulation or real robot experimentation. Currently, PnC uses the PyBullet simulator (Coumans and Bai, 2016–2019).

RobotSystem: The StateEstimator class computes the floating base state estimation using SensorData and updates the internal robot model using RobotSystem class. RobotSystem is a wrapper class for rigid body dynamics, such as interfacing with Dart (Lee et al., 2018) or Pinocchio (Carpentier et al., 2019). The class is initiated with a robot description file, updates the robot’s configuration, and implements APIs that return the robot’s kinematics and dynamics information, such as link Jacobians and the robot’s mass matrix.

ControlArchitecture: This is the main class that computes Commands, and it is composed of StateMachine, TaskContactContainer, and IHWBC. StateMachine includes a finite number of states, where each one determines distinct planner or controller parameter. State transitions are triggered by predefined temporal parameters, contact events, or user interrupts through the InterruptLogic class. At each state in the state machine, the TrajectoryManager communicates with the Planner and updates the desired task accelerations ${\ddot{x}}_{i}^{d}$ and reaction wrenches $f_{r}^{d}$. The HierarchyManager updates the implicit hierarchy between the tasks by modifying the task weights *w*_*i*_, and the ReactionForceManager updates the maximum reaction force $f_{r}^{\text{max}}$. Note that continuous changes to *w*_*i*_ and $f_{r}^{\text{max}}$ ensure smooth task and contact transitions. The TaskContactContainer contains a list of Tasks and Contacts that are used in IHWBC and which provide APIs for returning the quantities required to solve the QP optimization described in Eq. 5. PnC provides a predefined task and contact library that can be reused in other WBC implementations thus allowing flexible use for different applications. Currently, four tasks and two contact types are provided in the library: joint position task, Cartesian position task, orientation task, COM task, point contact, and surface contact. IHWBC solves a QP optimization and integrates the resulting joint accelerations to compute *τ* ^cmd^, *q* ^cmd^, and ${\dot{q}}^{\text{cmd}}$, which are then sent to the robot using the Command class.

## 5 Results

In this section, we evaluate our planning algorithm, TOWR+, and the whole-body control framework, IHWBC, with various robots in both simulation and real hardware. The simulation and experiment videos are available at https://youtu.be/XionNtDvM20.

### 5.1 TOWR+

We demonstrate a variety of motions generated with TOWR+ algorithm. We also study the effectiveness of our CRB model which employs the proposed centroidal inertia network. Finally, we perform a numerical complexity analysis of the TOWR+ algorithm.

#### 5.1.1 Illustrative Examples

Here, we consider various locomotion tasks with the NAO robot from SoftBank (Shamsuddin et al., 2011), Valkyrie from NASA (Radford et al., 2015), and ATLAS from Boston Dynamics. Before solving the trajectory optimization, we first trained a centroidal inertia network for each robot. To create the dataset, we followed the pipeline shown in Figure 4. We considered the robots’ kinematic limits and use them to sample a wide range of parameters so that the dataset includes configurations for many types of locomotion. The probability distribution used for sampling can be found in the open-source code repository we mentioned earlier. We generated 10^4^ end-effectors’ motions and then collected 15 data points per motion. We built the centroidal inertia network using a multi-layer perceptron with two hidden layers containing 64 nodes and tanh activation functions for differentiability. Finally, we obtained an analytic expression of the trained regressor and its Jacobian with respect to the inputs using the CasADi tool (Andersson et al., 2019).

**FIGURE 6:**
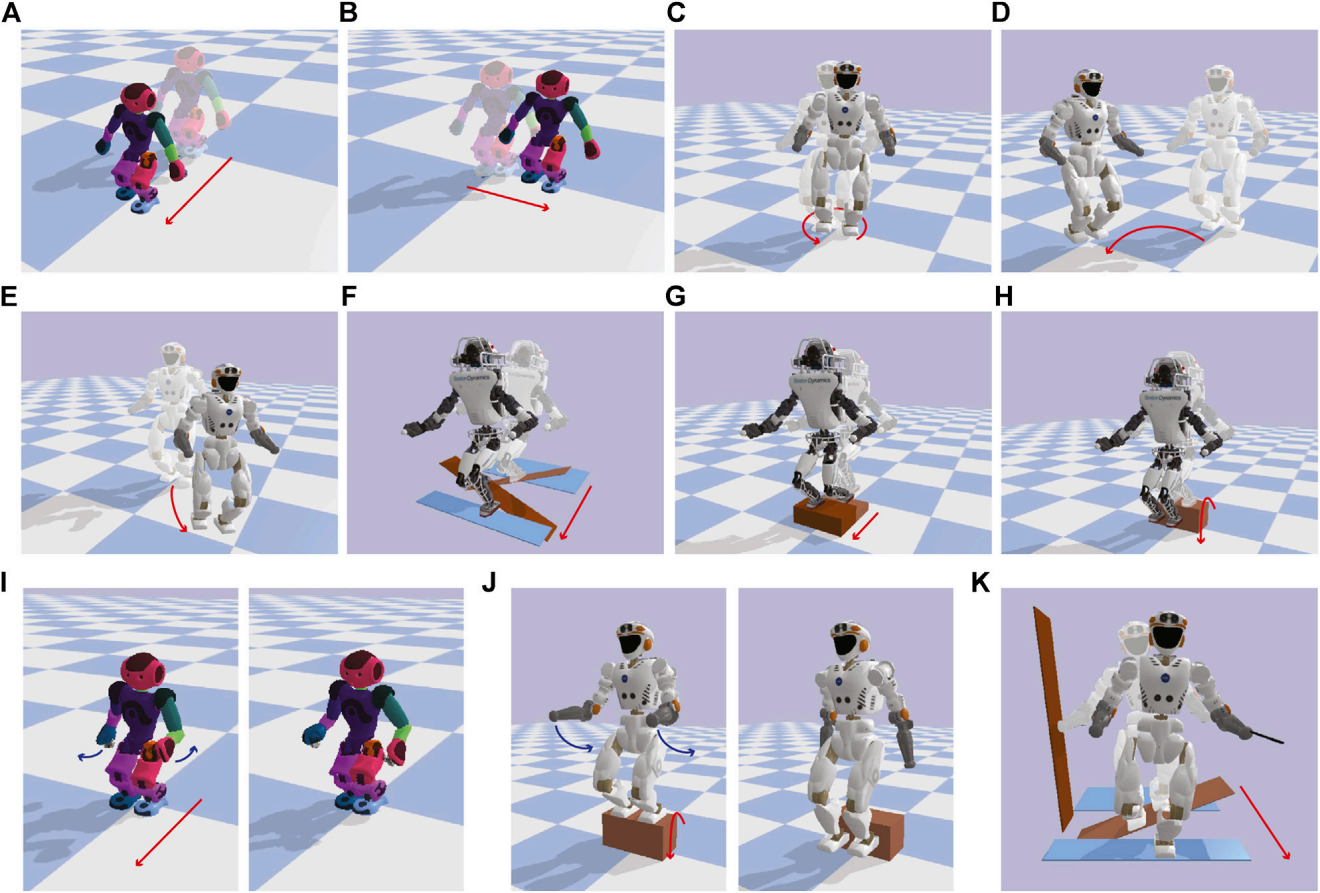
Various motions generated with TOWR+ using different robots are illustrated; **(A)** Forward walking, **(B)** side walking, **(C)** turning, **(D)** side walking while turning, **(E)** forward walking while turning, **(F)** cluttered terrain running, **(G)** stair climbing, and **(H)** hopping down a block. **(I)** and **(J)** illustrate forward walking and hopping down behaviors, while performing arm motion optimizations. **(K)** demonstrates cluttered terrain walking of Valkyrie while making multi-contact motion using its hands. For visualization we solve the robot’s inverse kinematics using the trajectories computed by TOWR+ and display the resulting joint motions.

Equipped with the trained centroidal inertia network, we generated various motions on the NAO, Valkyrie, and ATLAS moving in different terrains, as shown in Figure 6. For each example, we specified an initial and final state of the system, total duration of the motion, the number of contacts for each end-effector, and terrain information to generate a desirable walking trajectory. More detailed problem specifications including terrain information can be found in the provided code repository. We then solved for the base motion trajectories, [*r*(*t*), *θ*(*t*)], each end-effectors’ motions [*p*_*i*_(*t*), *ϕ*_*i*_(*t*)] and wrench trajectories [*f*_*i*_(*t*), *τ*_*i*_(*t*)], as well as the time duration for each phase (Δ*T*_*i*_). As shown in Subfigures 6A to 6H, our TOWR+ motion planner generated various locomotion behaviors including running over a cluttered terrain and jumping over a ground block by optimizing the phase duration vectors. By including the arms in the optimization process, TOWR+ discovered new arm motions that modify the robot’s composite inertia as shown in Subfigures 6I and 6J. For instance, the NAO robot adapted its centroidal inertia using its arms such that the volume of its inertia ellipsoid changed by 13% during forward walking (Figure 6I) compared to the case without arm motions (Figure 6A). To the best of our knowledge, inertia shaping capabilities using arm motions used for complex terrain locomotion planning have not been achieved by other simplified model-based motion planners. Finally, TOWR+ successfully produced motion trajectories to handle multi-contact scenarios as shown in Figure 6K.

For all examples in Figure 6, we discretized the base motion trajectories with a 0.1 s timestep. We used two polynomials to represent the foot motions during the swing phase and three polynomials for the contact wrench profiles during the stance phase. We enforced kinematic reachability constraints every 0.08 s and dynamics constraints at every 0.1 s. We imposed the terrain elevation constraint and the contact wrench cone constraint only at the junctions of the feet polynomials, but these constraints could be enforced with a finer granularity. For the stair and the block shown in Figures 6G,H,J, we smoothly increased or decreased the height around the edges with a small margin so that the solver could access the gradient information of the terrain. Finally, we solved the optimization problem using the Interior point method solver, Ipopt (Winkler, 2018).

#### 5.1.2 Quantitative Analysis

**FIGURE 7:**
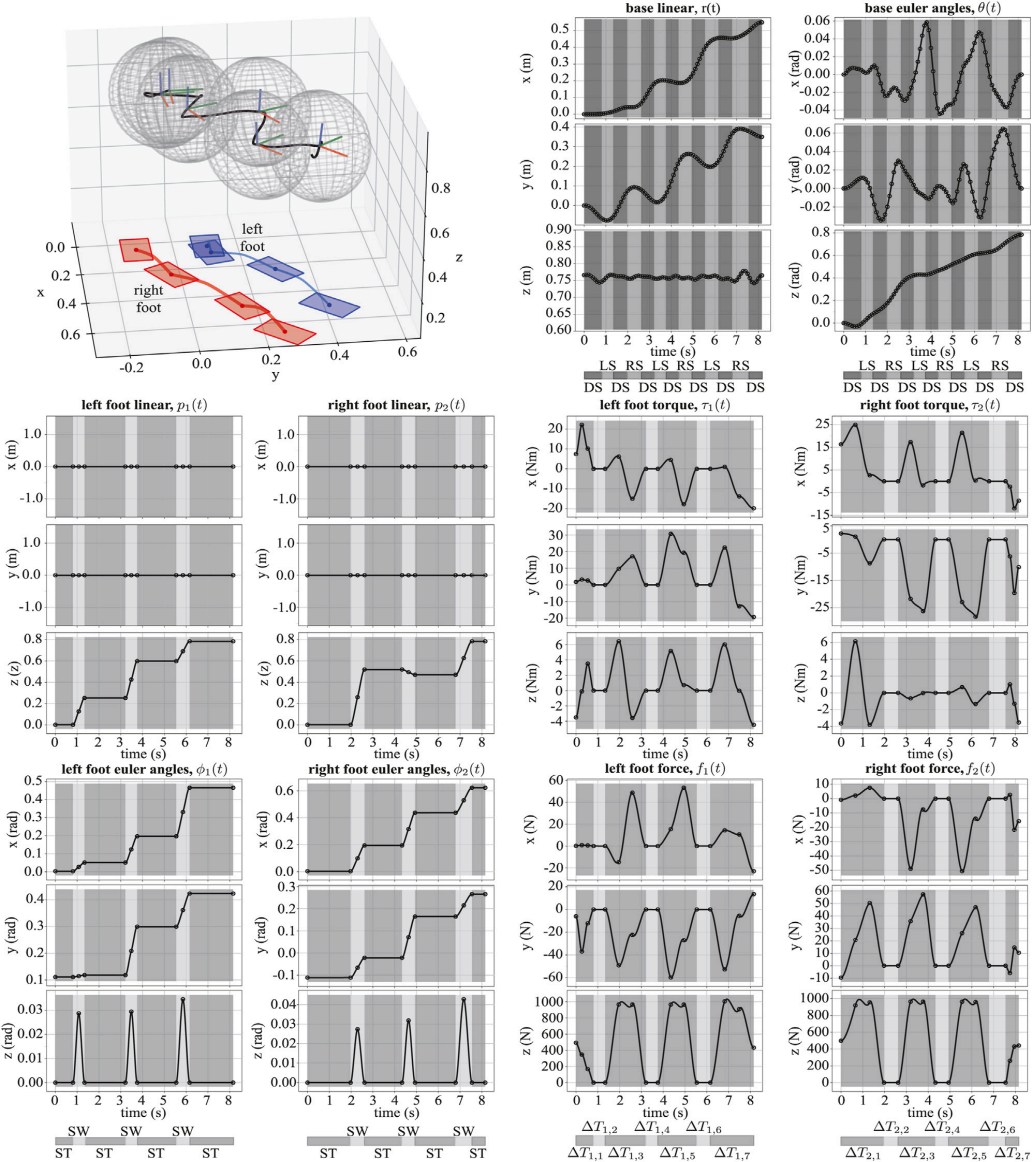
We illustrate the trajectories for Valkyrie walking forward while turning. The upper left figure shows the motions in Cartesian space with varying centroidal inertia ellipsoids.

First, we study solution trajectories for the example shown in Figure 6E. The solution trajectories for this example are shown in Figure 7. As mentioned, the optimization nodes for the base motions are equally distributed with a 0.1 s interval, whereas the nodes for the feet motions and wrenches are defined based on the contact phase. For instance, the feet motions in swing mode and the wrench profiles in stance mode are represented with two and three polynomials respectively. At the same time, a single polynomial with a constant value is used to represent the stance phase of the feet. Another polynomial with zero value is used to represent the wrench trajectories during the swing phase. This strategy ensures that the feet do not slip during contact and do not generate wrenches in the air. The gait pattern and schedule are also optimized using the phase duration variables, Δ*T*_1_ and Δ*T*_2_.

We now evaluate the centroidal inertia network during the walking and turning behavior shown in Figure 6E. At every 0.1 s, we solved the inverse kinematics problem using the output trajectories and computed ground truth centroidal inertia tensor and angular momentum rate. Then, we computed the average root-mean-squared percentage errors for the robot’s inertia and angular momentum rate when using our network’s predictions with respect to ground truth values. For comparison, we also implemented TOWR+ with a constant centroidal inertia tensor computed at a nominal pose instead of using the composite centroidal inertia network, and computed the previous errors again. As shown in Table 2, the centroidal inertia network provides much more accurate predictions, which means that the solution trajectories are achieved with higher fidelity.

**TABLE 2:** Average root-mean-squared percent errors of the centroidal inertia (*I*_*xx*_, *I*_*yy*_, *I*_*zz*_) and angular momentum rate ($\dot{L}$) predictions when using our trained our centroidal inertia network and a constant inertia computed at a nominal pose.

	***I*_*xx*_**	***I*_*yy*_**	***I*_*zz*_**	$\dot{L}$
Centroidal inertia network	1.05 (*%*)	0.43 (*%*)	0.114 (*%*)	0.99 (%)
Constant inertia at nominal pose	10.69 (*%*)	9.41 (*%*)	8.05 (*%*)	8.91 (%)

We also study computational efficiency of our centroidal inertia network by measuring computation time to evaluate our network and its Jacobian during the optimization process. We take the example with Valkyrie shown in Figure 6E. The evaluation of the regressor function and its Jacobian took place at 8,216 and 39,840 times during the optimization and took 0.37 and 5.58 s, respectively. To compare this, we solve the robot’s inverse kinematics (Buss, 2004) to obtain joint positions and then obtain the centroidal inertia instead of using our pre-trained centroidal inertia. With the inverse kinematics method, the total evaluation time of the centroidal inertia tensor and its Jacobian took 49.72 and 4,302.72 s, respectively. Note that we provided an analytic expression of the regressor network and its Jacobian to the solver, but in the inverse kinematics approach, we had to use the numerical Newton-Raphson method and the finite difference method.

**FIGURE 8:**
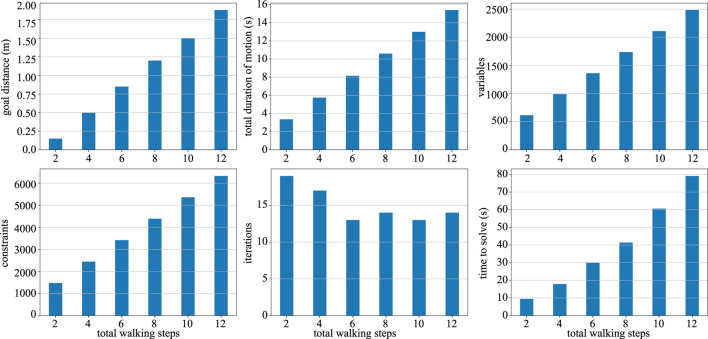
Complexity analysis for TOWR+ is shown here for six different forward walking behaviors each defined with a different specification. As the complexity of the problem increases linearly (i.e., the goal distance, total duration, and number of steps), the number of decision variables and constraints increases linearly, while the wall-clock time to solve the problem increases almost linearly.

Finally, we perform a numerical complexity analysis of our trajectory optimization framework. As illustrated in Figure 8, we defined six different forward walking behaviors with different goal distances, total duration of the motion, and total number of steps. For each case, we counted the number of decision variables, constraints, optimization iterations with respect to the wall-clock time. As the complexity increases linearly, the number of decision variables and constraints increases linearly too, whereas the wall-clock time to solve the problem increases almost linearly. The number of iterations to find a solution remains the same, regardless of process complexity during forward walking tasks. The numerical computations performed in this section were run on a single core of an Intel I7-4770HQ at 2.5 GHz.

### 5.2 IHWBC

**FIGURE 9:**
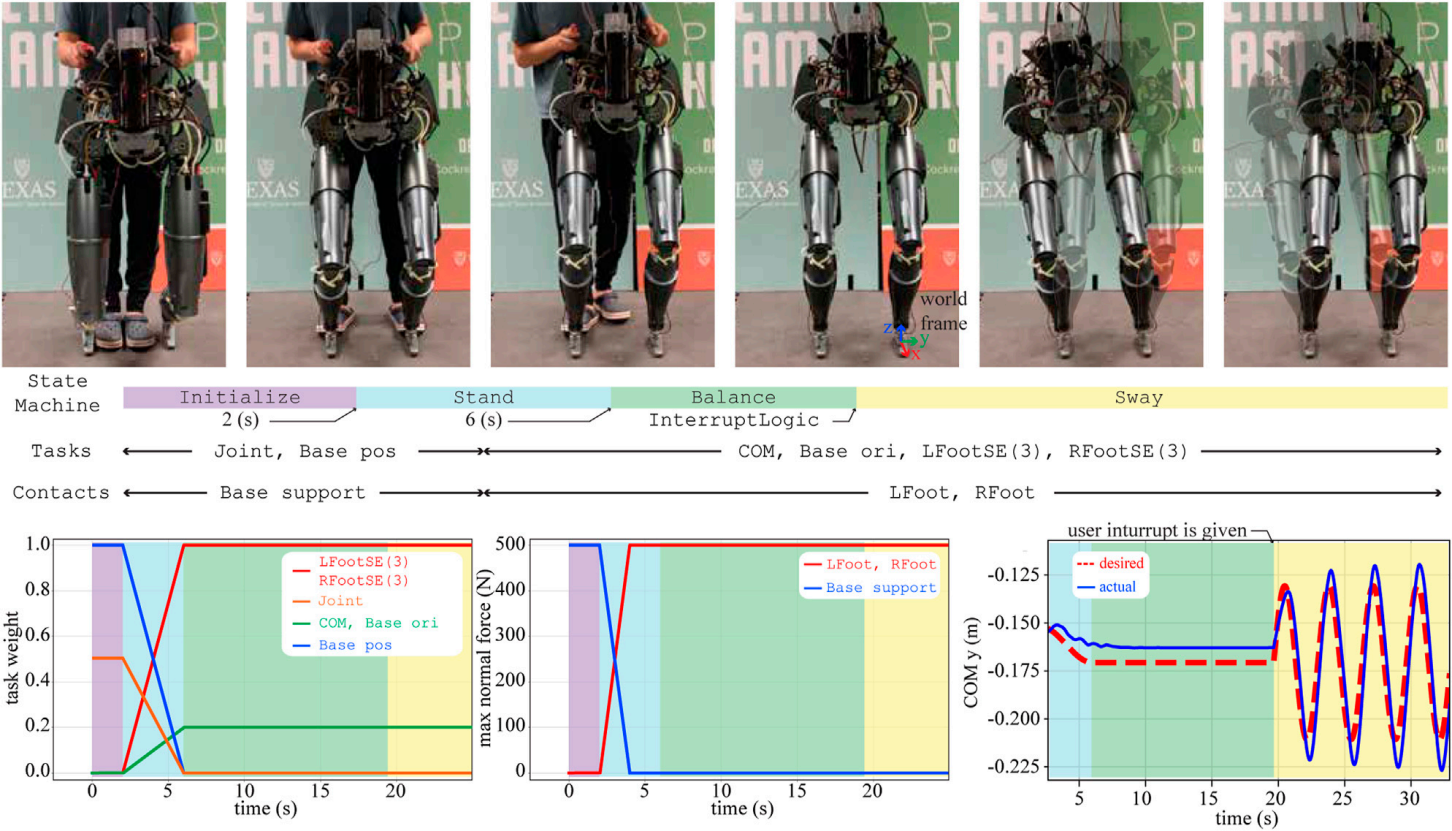
A sequence of snapshots of the balancing experiment are shown. Different sets of tasks and contacts are considered for each behavior phase. Using task weights we impose unilateral contact constraints and *implicit hierarchies*. The maximum normal forces (i.e., $f_{r}^{\text{max}}$ in Eq. 5) during the Stand phase are smoothly changed. The lateral COM trajectories (i.e., COM y) are also illustrated.

Here, we tested our biped DRACO robot balancing using IHWBC. As shown in Figure 9, the StateMachine class that we used consists of a sequence of six different states to achieve the desired balancing behavior.

Initialize: DRACO is initialized in the air where its base (i.e., torso) is supported by an overhead gantry with guidance from the experimenter. During the Initialize phase, DRACO moves its joints to a predefined target configuration for standing by employing reaction forces with the support ground. To be more specific, IHWBC controls the 10-dimensional joint positions and the 3-dimensional base position using a 3-dimensional reaction force from the base support contact. The desired base motions are controlled with zero accelerations with a high task weight to be fixed, while the desired joint motions are controlled using an interpolation between initial and target configurations. The Initialize phase lasts for 2 s and then switches to the Stand phase.

Stand: In this phase, DRACO uses the reaction forces from the ground to lift itself up. During this phase, IHWBC performs a smooth task transition from one task set (i.e., joint task and base position task) to another task set [i.e., 3-dimensional COM task, 3-dimensional base orientation task, 6-dimensional left and right foot SE(3) tasks] as well as a continuous contact transition from one contact set (i.e., base support contact) to another contact set (i.e., 6-dimensional left and right foot contacts^6^). The smooth changes of the task weights and maximum normal force in Figure 9 ensure continuous task and contact transitions. Next, the desired trajectories for the COM are determined by interpolating between initial and target positions, where the target *x* and the *y* position correspond to the middle of the feet and the target height *z* is set to be 0.7 m. The desired orientation for the base frame is set to be identical to the world frame. The desired accelerations for the feet motion tasks are set to zero to ensure the fulfillment of unilateral contact constraints. The Stand phase lasts for 4 s and switches on to the Balance phase.

Balance: During this phase, DRACO balances without any support from the overhead gantry or experimenter. During this phase, IHWBC maintains the COM and base orientation steady and forces the feet accelerations to become zero to prevent slipping. There are no contact forces from the handles due to experimenter interactions, and the feet control their reaction wrenches for balancing. The Balance phase lasts until receiving a user interrupt *via* InterruptLogic and then switches to the Sway phase.

Sway: In this state, the desired lateral motions for the COM task are determined using a sinusoidal trajectory planner using 0.04 m for the amplitude and 0.3 Hz for the frequency. The other tasks and contacts are kept identical to those from the previous Balance phase. The tracking performance in the lateral direction is shown in Figure 9.

During this balance experiment, we determined the desired joint accelerations using IHWBC and integrated them to provided the desired joint position and velocity commands to send to the low-level controller of the robot.

### 5.3 End-to-End Evaluation

Here, we integrate our TOWR+ and IHWBC algorithms and evaluate the full PnC framework both in simulation and in hardware experiments.

#### 5.3.1 ATLAS Walking With Unknown Disturbances

**FIGURE 10:**
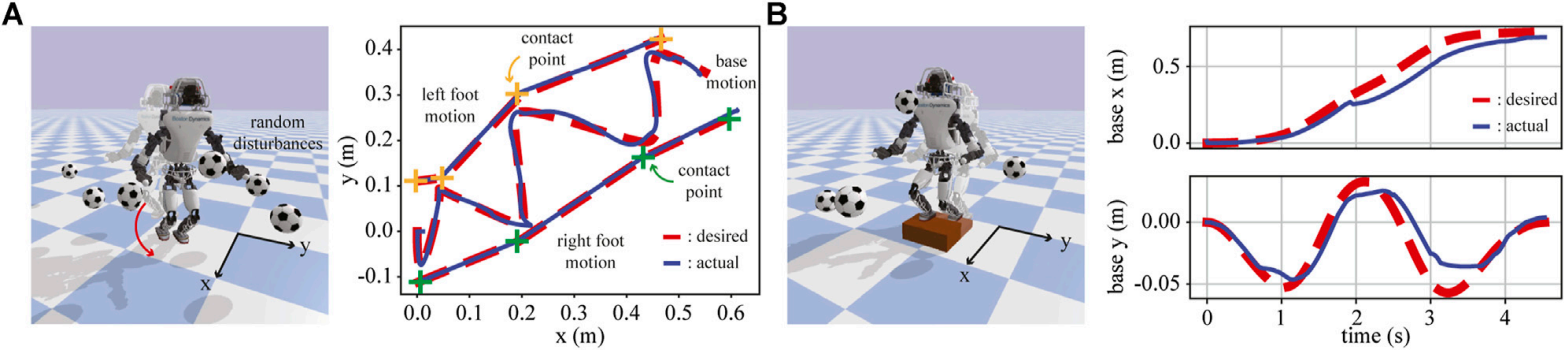
IHWBC stabilizes the robot along the solution trajectory computed by TOWR+ under unknown disturbances in the physics simulator. **(A)** ATLAS walking with a top view of its walking trajectories, and **(B)** ATLAS climbing a stair with its base trajectories being illustrated.

We compute ATLAS walking trajectories using TOWR+ and stabilize the robot using IHWBC in the physics simulator, PyBullet (Coumans and Bai, 2016–2019). As shown in Figure 10, we consider a case involving forward walking while turning and another case involving stair climbing. While controlling the robot, we generated random disturbances by throwing a 500 g soccer ball every 1 s. IHWBC rejected successfully the disturbances and achieved robust locomotion behaviors without falling.

#### 5.3.2 DRACO Step-and-Stop Experiment

**FIGURE 11:**
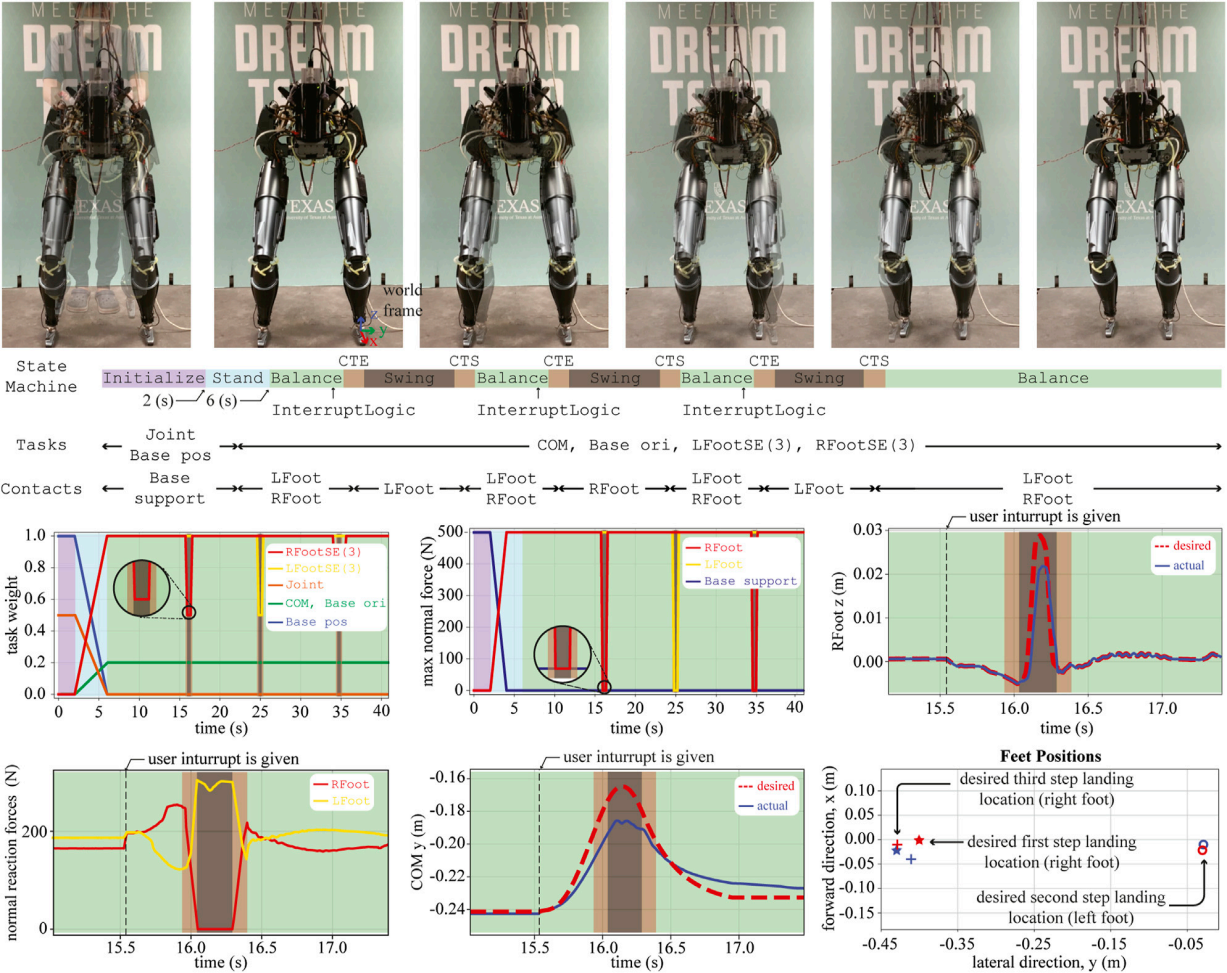
A sequence of snapshots for the step-and-stop experiment are shown. Different sets of tasks and contacts are considered for each behavior phase. Similarly to the balancing experiment, we show the task weights and the maximum normal forces throughout the experiment. The motion in the vertical direction of the swing foot (i.e., RFoot z) and the lateral COM trajectories (i.e., COM y) are also shown. The normal direction of the reaction force commands (i.e., $f_{r}^{*}$) at both feet computed by IHWBC are also shown. Finally, three sets of desired and actual foot landing locations during the experiments are depicted, where the red and blue color represent the desired and actual positions, respectively.

In this section, we integrate TOWR+ and IHWBC to demonstrate step-and-stop behaviors using the DRACO biped. Figure 11 shows three consecutive step-and-stop motions. Similarly to the balance experiment, we initialize the robot to the Balance phase. Then, an InterruptLogic (e.g., keyboard stroke) initiates TOWR+ to generate step-and-stop trajectories. Here, the robot swings either its left or right foot and stops at the nominal standing pose for 1.2 s. Once a solution is achieved by TOWR+, phase transitions are triggered based on the phase duration while the robot is stabilized along the trajectory using IHWBC.

ContactTransitionEnd (CTE): In this phase, we take off a small portion (i.e., 0.1 s) of the last part of the double support phase (i.e., Balance phase) and modify it to achieve smooth task and contact transitions. More specifically, CTE employs feet motion tasks, COM position task, and base orientation task, as well as feet contacts. Here, we continuously reduce the task weight and maximum normal force for each foot about to start swinging so that the robot can smoothly break contact. In this transition phase, the desired accelerations for the swinging foot are still zero, but the unilateral contact constraint becomes weaker as the task weight decreases. Similarly, IHWBC reduces the reaction forces from the swinging foot *via* reducing the maximum normal force $f_{r}^{\text{max}}$. The task weights and the maximum normal force during this phase are illustrated in Figure 11.

Swing: In this phase, the robot swings its leg using the trajectories computed by TOWR+. The phase employs the same type of tasks then CTE but with an implicit priority order as follows: contact foot motion task, swing foot motion task, COM task, and base orientation task. During step-and-stop motions, the desired foot and COM trajectories are shown in Figure 11. We also illustrate the desired normal direction of the contact reaction forces computed by IHWBC during swinging.

ContactTransitionStart (CTS): In this phase, we take off a small portion (i.e., 0.1 s) of the double support phase (i.e., the Balance phase) and modify it to achieve smooth task and contact transitions. CTS employs the same tasks and contacts as in the Swing phase but continuously increasing the task weight and maximum normal force of the swinging foot so that the robot can smoothly make contact. The task weights and max normal force during this phase are illustrated in Figure 11.

In summary, we have incorporated the proposed TOWR+ and IHWBC algorithms to achieve step-and-stop motions with DRACO. Thanks to the use of the CRB model in TOWR+, we have been able to plan dynamic motion trajectories employing accurate centroidal inertia information compared to other simplified model-based planners, such as the inverted pendulum-based planner. In addition to this, our IHWBC method provides flexibility for multi task management and smooth transitioning capabilities for step-and-stop motions resulting in small foot placement errors (less than 0.036 m as shown in Figure 11). To the best of our knowledge, stepping behaviors on a line-foot biped with significant leg mass and centroidal inertia variations has not been achieved before. Although this is an achievement of TOWR+ and IHWBC, we admit that there is a gap between the simulations with other robots and the DRACO step-and-stop experiment. In the following section, we discuss hardware limitations that hinder simulation like behaviors and future research directions that can possibly push forward the limits.

## 6 Discussion

In this paper, we have proposed a trajectory optimization framework, TOWR+, and a whole-body control formulation, IHWBC, plus their implementation in an open-source code repository, PnC. TOWR+ employ a novel composite rigid body inertia model, CRB, to model humanoid robot dynamics more accurately than simpler centroidal dynamic model. In addition, it incorporates extensions to deal with flat feet contacts including 6D reaction wrenches and SE(3) feet trajectories. Like TOWR, TOWR+ does not rely on integer programming or complementarity constraints.

We have demonstrated various advanced locomotion behaviors on different humanoid robots and conducted a thorough analyses of complexity of TOWR+. For feedback whole-body control, we have devised a new algorithm, IHWBC, which establishes implicit hierarchies between multiple tracking tasks and defines soft constraints to deal with unilateral contact constraint. This method allows for smooth task and contact transitions and provides versatility and ease-of-use for general biped robots and humanoids. Finally, we have integrated the proposed TOWR+ novel trajectory optimization method and IHWBC to demonstrate balancing and a step-and-stop behaviors in our real DRACO biped robot.

Although the step-and-stop experiment has been implemented in the real hardware, there are a few hardware limitations on Draco that hinder more complex demonstrations. Due to the small line feet and the comparatively heavy distal mass, the dynamic stability of the robot requires quick swing motions (<0.3 s) and a small swing height (<3 cm). However, the physical robot has a motion bandwidth that is upper bounded by the mechanical linkage structures and the actuation power. In the step-and-stop experiment, we were able to control the stepping motion within a 3.6 cm error using IHWBC, which is enough for one step-and-stop motion but not for continuous walking.

With that in mind, we consider a few future research directions. Although TOWR+ is computationally efficient in discovering novel contact sequences and generating locomotion trajectories, it is not fast enough to be implemented as a receding horizon controller, which is necessary for the robust continuous walking. We are interested in devising generative models to provide a good initial guess for warm starting TOWR+. Finally, we are looking forward to deploying the proposed end-to-end framework in our upcoming humanoid robot which has biped legs, with 6 DOF for each of them, and a two arm manipulation torso with an articulated head equipped with a vision sensor.

## Data Availability

The datasets presented in this study can be found in online repositories. The names of the repository/repositories and accession number(s) can be found below: https://github.com/junhyeokahn/PyPnC.

## Footnotes

1 The properties of the robot are taken from the description files in the following repositories: https://github.com/ANYbotics/anymal_b_simple_description (ANYmal B), https://github.com/erwincoumans/pybullet_robots (Cassie, ATLAS), https://github.com/gkjohnson/nasa-urdf-robots (Valkyrie), and https://github.com/junhyeokahn/PnC (DRACO).

2 Exceptionally, Cassie has the leg actuators carefully located near the torso, which results in less variation of centroidal inertia being an atypical humanoid.

3
https://github.com/junhyeokahn/PnC

4
https://github.com/junhyeokahn/PyPnC

5 Given pairs of foot positions, *x*_0_ and *x*_1_, and their derivatives, ${\dot{x}}_{0}$ and ${\dot{x}}_{1}$, at the end points with time duration Δ*T*, a polynomial can be fully defined as *x(t*) = *a*_0_ + *a*_1_ *t* + *a*_2_ *t* ^2^ + *a*_3_ *t* ^3^, where $a_{0}=x_{0},\;a_{1}={\dot{x}}_{0},\;a_{2}=-\Delta T^{-2}\left(3\left(x_{0}-x_{1}\right)+\Delta T\left(2{\dot{x}}_{0}+{\dot{x}}_{1}\right)\right)$, and $a_{3}=\Delta T^{-3}\left(2\left(x_{0}-x_{1}\right)+\Delta T\left({\dot{x}}_{0}+{\dot{x}}_{1}\right)\right)$.

6 DRACO’s line-foot is approximated using a narrow surface contact.
